# Male involvement in PMTCT and associated factors among men whom their wives had ANC visit 12 months prior to the study in Gondar town, North west Ethiopia, December, 2014

**DOI:** 10.11604/pamj.2016.24.239.8460

**Published:** 2016-07-14

**Authors:** Abdella Amano, Abdulbasit Musa

**Affiliations:** 1 School of Public and Environmental Health, College of Medicine and health sciences, Hawassa University, Ethiopia; 2 Department of Midwifery, College of Health and Medical sciences, Haramaya University, Ethiopia

**Keywords:** Male involvement, PMTCT, Gondar, Ethiopia

## Abstract

**Introduction:**

Globally, male involvement has been recognized as a priority focus area to be strengthened in PMTCT but, testing male partners for HIV in the context of preventing mother-to-child transmission remains a challenge in most low- and middle-income countries including Ethiopia. In Ethiopia even though male involvement is one of the guiding principle in testing and counseling of HIV, the magnitude of male involvement in PMTCT is not well known. The objective was to assess the magnitude of male involvement in PMTCT and associated factors among men whom their wives have ANC visit 12 months prior to the study in Gondar town, North west Ethiopia.

**Methods:**

A community-based cross-sectional survey was conducted from December 1- 20, 2014 among men whose wives had ANC follow up in the last 12 months prior to study period in Gondar town. Cluster sampling was used to get the total of 802 participants.

**Results:**

From all participants, only 20.9% of men had high involvement index in prevention of mother to child transmission of HIV/AIDS. Men with secondary and post secondary education (AOR=3.59, 95%CI: 1.36, 9.44), government employment by occupation (AOR=2.23, 95%CI: 1.53, 4.02) men who were married and in union (AOR=4.37, 95%CI: 1.85, 10.32), and men who have heard about PMTCT (AOR=1.74, 95%CI=1.21, 2.49) were more likely to have high involvement index in PMTCT.

**Conclusion:**

Male involvement in PMTCT programme was low in the study area. Having information about PMTCT, attending Secondary and post secondary education, being government employer and living in union with partner were factors significantly associated with male involvement in PMTCT. Improving male involvement by creating husband’s awareness regarding benefit of PMTCT through provision of balanced information for all male partners is recommended.

## Introduction

Human Immunue Deficiency Virus (HIV) is transmitted from an HIV-infected mother to her child during pregnancy, labor, delivery or breastfeeding is known as mother-to-child transmission (MTCT) and preventing HIV transmission in pregnant women and their children often referred to as prevention of mother-to-child transmission (PMTCT) [1, 2]. These PMTCT become a crucial intervention in the global fight against this epidemic [2]. To prevent the transmission of HIV from mother to baby, the World Health Organization (WHO) promotes a comprehensive approach, which include preventing HIV transmission from a woman living with HIV to her infant [3, 4]. An estimated 430,000 children were newly infected with HIV in 2008, over 90% of them through mother-to-child transmission (MTCT). Without treatment, about half of these infected children will die before their second birthday [5]. MTCT is the primary cause of all HIV infections in children under fifteen years of age [6]. Without intervention, the risk of MTCT ranges from 20% to 45%. With specific interventions in non-breastfeeding populations, the risk of MTCT can be reduced to less than 2%, and to 5% or less in breastfeeding populations [7, 8]. Globally, male involvement has been recognized as a priority focus area to be strengthened in PMTCT [8] but testing male partners for HIV in the context of preventing mother-to-child transmission remains a challenge in most low- and middle-income countries. In 2008, 57 countries documented the number of male partners of pregnant women attending antenatal care who received an HIV test. The proportion of pregnant women attending antenatal care whose male partners were tested for HIV was 5% in 2008 versus 2% in 2007 [9].

Studies have shown that the utilization of PMTCT services by the pregnant women is influenced both by factors related to the health system such as accessibility of VCT services, and by individual factors such as fear of disclosure of HIV results, lack of male partner support, fear of domestic violence, abandonment and stigmatization [2, 5, 10]. Couple VCT was shown to have greater benefits than accompanying the female partner for individual VCT [11]. Unfortunately, few men accompany their partners to antenatal clinics and even fewer participate in couple counseling when it is available [12, 13]. There is evidence that lack of partner support is associated with poor uptake of antiretroviral medication and the inability to modify infant feeding practices [7, 14]. Sexual abstinence and condom use have also been shown to be more common among postpartum women who reveal HIV-positive results to partners [15, 16]. These associations between partner involvement and uptake of interventions underscore the importance of involving the male partner in HIV-1 prevention efforts initiated in the antenatal setting [17]. Low rates of HIV testing among women in antenatal setting have several implications for PMTCT programmes as the optimal uptake and adherence to such programmes is difficult for women whose partners are either unaware or not supportive of their participation. So, to improve utilization of HIV testing among women in ANC, assessing magnitude of male involvement in PMTCT and associated factors will have important implications to address the perspective issues.

## Methods

A community based cross-sectional study was conducted in Gondar town from December 1- 20, 2014. Gondar town is found in North Gondar zone of Amhara regional state and is located 750 km Northwest of Addis Ababa. According to the 2007 Ethiopian census report, Gondar has a total population of 206, 987 and adolescents aged 15-19 years are estimated to be (25,128) 12% of the total population. Administratively, the town is divided into 12 administrative areas. The health system in the town is represented by one referral hospital, eight health centers and two governmental youth center. In addition, there are five higher clinics, one hospital, twenty two medium clinics; nineteen lower clinic owned by private sectors and two NGO clinics. The study populations were men whose wives had ANC follow up in the last 12 months prior to study period in the selected clusters. Cluster sampling technique was used to select the study units. By taking admistrative area as cluster, 4 out of 12 admistrative areas were selected. Then, all eligible men in the selected administrative area were included in the study. The sample size was determined by using a single population proportion formula considering the following assumptions: Magnitude of male involvement in PMTCT 50% (**p**= 0.5) as there was no previous study, 5% level of significance (a = 0.05), 5% marginal error (**d**= 0.05). The final sample size was adjusted by using design effect of 2 and 5% non-response. Finally the sample size determined was 808. Data was collected by face to face interview using a structured and pre-tested questionnaire. Ten diploma nurses were used to collect data. Three Midwives from University of Gondar teaching Hospital were assigned to supervise the data collection process. Training was given for both data collectors and supervisors. The level of male involvement in PMTCT programme was measured using six questions adapted from similar study conducted in Uganda (17). The questions were modified to local context and include: whether the man attends antenatal care with his partner, whether the man knows the partner's antenatal appointment, whether the man discusses antenatal interventions with his Partner, whether the man supports his partner's antenatal visits financially, whether the man has sought permission to use a condom during the current pregnancy and whether the man tested for HIV with his partner. The involvement score for each respondent range from 0 = no involvement to 6 = involved in all 6 activities. A total score of **4-6** was considered as a **'high'** male involvement score and **0-3** as **'low'** male involvement. Data entry was done by using EPI Info 2002 and exported to SPSS version 20.0 soft ware package for analysis. The presence of association between independent and dependent variables was assessed using odds ratio with 95% confidence interval by applying logistic regression model. Ethical clearance was obtained from College of Medicine and Health Sciences ethical review committee, University of Gondar. Formal letter of cooperation was written for North Gondar Zone Health Department and Gondar woreda Health Office. After informing the objective of the study, consent was obtained voluntarily from each study subject.

## Results

### Socio demographic characteristics

A total of 802 male whom their wives had ANC follow up in the last 12 months were interviewed; out of these 641(79.9%) were Orthodox religion followers. The mean age of the respondents was 33.17 years with SD of 7.25 years. The majority, 701(87.4%) of the husbands were married. Five hundred thirteen (64.0%) of the husbands were attended secondary and post secondary education. Among the respondents 572(71.3%) of the husbands were self employer. Concerning wives educational status, 475(59.2%) attended secondary and post secondary education (Table 1).

**Table 1:** Socio-demographic characteristics of the study participants, Gondar Town North West Ethiopia, December, 2014

Variables	Frequency (%)
**Age of the husbands at interview**	
20-24	46(5.7)
25-34	442(55.1)
35-44	243(30.3)
45^+^	71(8.9)
**Marital status**	
Married	695(86.7)
Divorced/separated	107(13.3)
**Religion**	
Orthodox	641(79.9)
Muslim	112(14.0)
Others^+^	49(6.1)
**Educational status of the husband**	
Unable to read and write	66(8.2)
Primary Education	223(27.8)
Secondary and post secondary	513(64.0)
**Occupational status of the husband**	
Government employee	572(71.3)
Self employee	230(28.7)
**Economic Status**	
<1000ETB	149(18.6)
1000-1999ETB	215(26.8)
2000-2999ETB	202(25.2)
>/= 3000ETB	236(29.4)
**Number of children**	
No child	56(7.0)
1-4	710(88.5)
5^+^	36(4.5)

+ Protestant, Catholic

### Level of male involvement in PMTCT programmed

The level of male involvement in PMTCT was assessed using six variables shown in Table 2. Only 219 (27.3%) of the 802 men had attended ANC with their partners. One in three (33.9%) of the husbands knows ANC appointment of their wives. The majority of respondents 554(69.1%) had not asked their partners whether they could use condoms during sexual intercourse with them. Only 176(21.9%) of husbands had tested for HIV with their wives. Only one in five (20.9%) of the husbands had high male involvement index in PMTCT (Table 2).

**Table 2:** Level of male involvement in PMTCT programme Gondar Town, Gondar, North West Ethiopia, December, 2014

Variable	Response N (%)	
	Yes	NO
Ever attended ANC with partner	219(27.3)	583(72.7)
Knows partner's ANC appointments	272(33.9)	530(66.1)
Provides financial support to partner to attend ANC	193(24.1)	609(75.9)
Discusses with partner information or interventions given in ANC	181(22.6)	621(77.4)
Asked partner if he could use a condom	248(30.9)	554(69.1)
Tested HIV with partner	176(21.9)	626(78.1)
Over all high male involvement index	168(20.9)	634(79.1)

### Factors associated with Male Involvement in PMTCT Programme

As depicted in the bivariate models, male involvement in PMTCT was associated significantly with age of the husband, husband occupation, educational status of the husband, having heard about PMTCT and marital status. From the variables found to be significant in the bivariate analysis; educational status of husbands, husband occupation, having heard about PMTCT and marital status were found to be significantly associated with male involvement in PMTCT in multiple logistic regression analysis. Men who heard about PMTCT were 1.7 times morelikely to have high involvement index in PMTCT as compared to those who didn’t hear. Those who attended secondary and post secondary education were 3.6 times more likely have high involvement index in PMTCT than those who cannot read and write. Those who are married and in union were about 4.3 times more likely to have high involvement index than those who were widowed or separated. Men whose occupation was government employee were about 2 times more likely to be involved in PMTCT as compared to self employer (Table 3).

**Table 3:** Bivariate and multivariate analysis of factors associated with Male involvement in PMTCT among men whose wives had ANC follow up in the last 12 months in Gondar town, December -2014, Gondar, North West Ethiopia

Variables	Male Involvement in PMTCT index		COR(95%CI)	AOR(95%CI)
	Low	High		
Age of the husband				
20-30	273	99	1.90(1.34, 2.68)	^+^
31^+^	361	69	1	
Husband Occupation				
Government employee	428	144	2.89(1.82,4.59)	2.23(1.53,4.02)
Self employee	206	24	1	1
Ever Heard about PMTCT				
Yes	541	158	2.72(1.38,5.34)	1.74(1.21,2.49)
No	93	10	1	1
Husband Education				
Unable to read and write	61	5	1	1
Primary education	181	42	2.83(1.07,7.48)	2.77(1.02, 7.56)
Secondary education and above	392	121	3.77(1.48,9.58)	3.59(1.36,9.44)
Marital Status				
Married	533	162	5.12(2.20, 11.88)	4.37(1.85,10.32)
Divorced/separated	101	6	1	1
Number of Children				
No child	39	17	1.81(0.66,4.92)	^+^
1-4	566	144	1.05(0.45,2.45)	
5^+^	29	7	1	

+ Not significant in multivariate analysis

## Discussion

The study results revealed that only one in four men (20.9%) had high PMTCTC involvement index in the study area. This finding is similar with study report from Mekele town health facilities, Ethiopian in which 20% of men had high PMTCTC involvement index [17]. The finding is higher than study conducted in Nairobi antenatal clinic, Kenya in which only 15% of male involved in VCT with their wives [11]. But this finding is lower when compared with the study from eastern Uganda 26% and Addis Ababa, Ethiopia 28.1% [16, 18]. The difference between our findings and other studies might be due to the difference of socio-economical and accessibility and availability of health care. In this study we have found a number of factors associated with male participation in the PMTCT programme. They include having had information about PMTCT, Husband education, occupational status of the husband and marital status. Those men's who had prior information about PMTCT about 1.7 times more likely to have high PMTCT involvement index as compared to those who had no information. This finding is in line with the study finding of Mbeya Region, Tanzania in which lack of information about PMTCT hinders male involvement in PMTCT [19]. Similarly study conducted in Addis Ababa revealed that those men's who had knowledge on PMTCT were more likely to have high PMTCT involvement index [18]. The possible explanation for this might be having information about PMTCT will help the partners to know the benefit of PMTCT programme for themselves as well as their new born thereby increase their involvement. Men's who attended secondary education and above were more like to have high PMTCT involvement index than those who were unable to read and write. This finding is in agreement with other study conducted in Uganda [16] and Zambia [20] where level of men's education had significant association with involvement in PMTCT. These might be due to the fact that educated men's had better awareness about the benefits of preventive health care including PMTCT. They might also have higher receptivity to new health related information. This study also revealed that men's whose occupation was government employee were about 2 times more likely to involve in PMTCT as compared to self employer. This finding is similar with the study finding of Addis Ababa [18] where government employer were more likely to involve in PMTCT than private employer. Study conducted in Uganda [16] also showed occupation of the husband had significant association with male involvement in PMTCT. The possible explanation for this might be that those government employers were more educated and had more awareness about health related issues than private employers like daily laborer. Those men's who are married and in union were about 4 times more likely to have high involvement index than those who were widowed or separated. These finding is comparable with study conducted in northern Tanzania where partners living together were more likely involved in PMTCT [21]. These might be due the fact that partners who live together had a penchant to discuss about health and related issue that can increase men's involvement in PMTCT.

## Conclusion

In conclusion, this study revealed that the proportion of men who had high PMTCT involvement index in the study area was low. Having had information about PMTCT, Secondary and post secondary education, being government employer and living in union with partner were factors significantly associated with male involvement in PMTCT. Improving male involvement by creating husband’s awareness regarding benefit of PMTCT through provision of balanced information for all male partners is recommended.

### What is known about this topic

- Magnitude of male involvement in PMTCT is low;
- Known factors for high PMTC involvement index was having had information on PMTCT and being employed.

### What this study adds

- Attending secondary and above level of education, and being married contribute to high PMTC involvement index.
